# Supplementary material for: COZOID: contact zone identifier for visual analysis of protein-protein interactions
Source: BMC Bioinformatics. 2018 Apr 6;19:125. doi: 10.1186/s12859-018-2113-6 (PMC5889581; doi:10.1186/s12859-018-2113-6)
Supplement: Supplementary file 2 — Software build. Executable binary file of the software tool. (ZIP 118,784 kb) [file 12859_2018_2113_MOESM2_ESM.zip › cozoid/java/docs/beansbinding-1.2.1-doc.zip!/org/jdesktop/el/impl/util/MessageFactory.html]

MessageFactory (Beans Binding)


---


|  |  |  |
| --- | --- | --- |
| **Overview** \| **Package** \| **Class** \| **Use** \| **Tree** \| **Deprecated** \| **Index** \| **Help** | |  |
| PREV CLASS   **NEXT CLASS** | **FRAMES**    **NO FRAMES**     **All Classes** |  |
| SUMMARY: NESTED \| FIELD \| CONSTR \| METHOD | DETAIL: FIELD \| CONSTR \| METHOD |  |


---


## org.jdesktop.el.impl.util Class MessageFactory

```
java.lang.Object
  org.jdesktop.el.impl.util.MessageFactory
```

---

``` public final class MessageFactory extends java.lang.Object ```

---

| **Field Summary** | |
| --- | --- |
| `protected static java.util.ResourceBundle` | `bundle` |


**Constructor Summary**

`MessageFactory()`


| **Method Summary** | |
| --- | --- |
| `static java.lang.String` | `get(java.lang.String key)` |
| `static java.lang.String` | `get(java.lang.String key, java.lang.Object obj0)` |
| `static java.lang.String` | `get(java.lang.String key, java.lang.Object obj0, java.lang.Object obj1)` |
| `static java.lang.String` | `get(java.lang.String key, java.lang.Object obj0, java.lang.Object obj1, java.lang.Object obj2)` |
| `static java.lang.String` | `get(java.lang.String key, java.lang.Object obj0, java.lang.Object obj1, java.lang.Object obj2, java.lang.Object obj3)` |
| `static java.lang.String` | `get(java.lang.String key, java.lang.Object obj0, java.lang.Object obj1, java.lang.Object obj2, java.lang.Object obj3, java.lang.Object obj4)` |
| `static java.lang.String` | `getArray(java.lang.String key, java.lang.Object[] objA)` |

**Methods inherited from class java.lang.Object**

`clone, equals, finalize, getClass, hashCode, notify, notifyAll, toString, wait, wait, wait`

**Field Detail**

### bundle

```
protected static final java.util.ResourceBundle bundle
```


**Constructor Detail**

### MessageFactory

```
public MessageFactory()
```


**Method Detail**

### get

```
public static java.lang.String get(java.lang.String key)
```

---


### get

```
public static java.lang.String get(java.lang.String key,
                                   java.lang.Object obj0)
```

---


### get

```
public static java.lang.String get(java.lang.String key,
                                   java.lang.Object obj0,
                                   java.lang.Object obj1)
```

---


### get

```
public static java.lang.String get(java.lang.String key,
                                   java.lang.Object obj0,
                                   java.lang.Object obj1,
                                   java.lang.Object obj2)
```

---


### get

```
public static java.lang.String get(java.lang.String key,
                                   java.lang.Object obj0,
                                   java.lang.Object obj1,
                                   java.lang.Object obj2,
                                   java.lang.Object obj3)
```

---


### get

```
public static java.lang.String get(java.lang.String key,
                                   java.lang.Object obj0,
                                   java.lang.Object obj1,
                                   java.lang.Object obj2,
                                   java.lang.Object obj3,
                                   java.lang.Object obj4)
```

---


### getArray

```
public static java.lang.String getArray(java.lang.String key,
                                        java.lang.Object[] objA)
```


---


|  |  |  |
| --- | --- | --- |
| **Overview** \| **Package** \| **Class** \| **Use** \| **Tree** \| **Deprecated** \| **Index** \| **Help** | |  |
| PREV CLASS   **NEXT CLASS** | **FRAMES**    **NO FRAMES**     **All Classes** |  |
| SUMMARY: NESTED \| FIELD \| CONSTR \| METHOD | DETAIL: FIELD \| CONSTR \| METHOD |  |


---
